# A risk score for identifying methicillin-resistant *Staphylococcus aureus* in patients presenting to the hospital with pneumonia

**DOI:** 10.1186/1471-2334-13-268

**Published:** 2013-06-06

**Authors:** Andrew F Shorr, Daniela E Myers, David B Huang, Brian H Nathanson, Matthew F Emons, Marin H Kollef

**Affiliations:** 1 Pulmonary and Critical Care Medicine, Washington Hospital Center, Washington DC, USA; 2 Pfizer Inc, Outcomes Research, Collegeville, USA; 3 Formerly of Pfizer Inc, US Medical Affairs, Collegeville, USA; 4 OptiStatim, LLC, Longmeadow, USA; 5 Cerner Corporation, Research Division, Culver City, USA; 6 Pulmonary and Critical Care Division, Washington University School of Medicine, St. Louis, USA

## Abstract

**Background:**

Methicillin-resistant *Staphylococcus aureus* (MRSA) represents an important pathogen in healthcare-associated pneumonia (HCAP). The concept of HCAP, though, may not perform well as a screening test for MRSA and can lead to overuse of antibiotics. We developed a risk score to identify patients presenting to the hospital with pneumonia unlikely to have MRSA.

**Methods:**

We identified patients admitted with pneumonia (Apr 2005 – Mar 2009) at 62 hospitals in the US. We only included patients with lab evidence of bacterial infection (e.g., positive respiratory secretions, blood, or pleural cultures or urinary antigen testing). We determined variables independently associated with the presence of MRSA based on logistic regression (two-thirds of cohort) and developed a risk prediction model based on these factors. We validated the model in the remaining population.

**Results:**

The cohort included 5975 patients and MRSA was identified in 14%. The final risk score consisted of eight variables and a potential total score of 10. Points were assigned as follows: two for recent hospitalization or ICU admission; one each for age < 30 or > 79 years, prior IV antibiotic exposure, dementia, cerebrovascular disease, female with diabetes, or recent exposure to a nursing home/long term acute care facility/skilled nursing facility. This study shows how the prevalence of MRSA rose with increasing score after stratifying the scores into Low (0 to 1 points), Medium (2 to 5 points) and High (6 or more points) risk. When the score was 0 or 1, the prevalence of MRSA was < 10% while the prevalence of MRSA climbed to > 30% when the score was 6 or greater.

**Conclusions:**

MRSA represents a cause of pneumonia presenting to the hospital. This simple risk score identifies patients at low risk for MRSA and in whom anti-MRSA therapy might be withheld.

## Background

Methicillin-resistant *Staphylococcus aureus* (MRSA) is an important pathogen in a number of conditions ranging from pneumonia to bacteraemia [1]. In critically ill patients, MRSA accounts for more than 70% of the *S*. *aureus* isolated [1,2]. Because of the multitude of infections associated with MRSA along with its potential severity, this pathogen results in substantial morbidity and mortality [1].

MRSA has often been implicated as a cause of either hospital-acquired or ventilator-associated pneumonia (HAP or VAP) [3]. In contrast, *Streptococcus pneumoniae*, *Haemophilus influenzae*, and *Legionella spp*. historically represent the most common bacterial causes of community-acquired pneumonia (CAP) [4]. However, a number of bacteria historically thought confined to nosocomial processes are increasingly isolated in community-onset infections. This trend fostered the creation of the concept of healthcare-associated pneumonia (HCAP) [3]. Although both patients with HCAP and CAP suffer the onset of their infections outside the hospital, those with HCAP are distinct because of their ongoing exposure to the healthcare system.

Unfortunately, reliance on the notion of HCAP to guide the use of broader spectrum antibiotics may result in overutilization of such agents. Moreover, HCAP as currently defined, appears to lack specificity as a screening test for resistant pathogens [5-7]. Prior studies evaluating risk factors for resistant pathogens in pneumonia presenting to the hospital suggest that grouping Gram-negative organisms such as *Pseudomonas aeruginosa* together with MRSA may be inappropriate [5-7]. These distinct pathogens possess different risk factors, or select risk factors for resistance may carry differential significance for the varying organisms. For example, theoretically, nursing home residence may be associated more with MRSA than *P*. *aeruginosa*[8]. Additionally, clinicians are able to either add or withhold anti-MRSA therapy without compromising the extent of the Gram-negative coverage. The decision to prescribe an anti-MRSA agent can be decoupled from the option of Gram-negative treatment. Several agents exist which possess only Gram-positive activity and are essentially employed only for their anti-MRSA properties. Thus, it seems prudent to better recognize the specific variables that are associated with an increased risk for MRSA. Understanding these patient characteristics would more precisely allow clinicians to segregate persons at risk for MRSA from those unlikely to be infected with MRSA. Furthermore, identifying those at low risk for MRSA would facilitate antibiotic stewardship by rationally limiting unnecessary anti-MRSA therapy which would help contain costs and reduce future antibiotic resistance [9]. Therefore, we conducted a retrospective analysis in order to develop a score to stratify patients presenting to the hospital with pneumonia by their risk for MRSA infection.

## Methods

### Study overview and patients

We conducted a multi-centre, retrospective analysis of patients admitted to the hospital with pneumonia between April 2005 and March 2009. The data are from 62 hospitals in the United States (US) via the Cerner *Health Facts* database (see Additional file 1). We included adults with a primary discharge diagnosis of pneumonia or a primary diagnosis of sepsis with a secondary diagnosis of pneumonia and no other source of infection by International Classification of Diseases, 9th Revision, Clinical Modification (ICD-9-CM) codes (see Additional file 2). The codes were selected based on existing published studies [10-12] in combination with clinical judgment. We excluded those younger than 18 years of age and individuals with only nosocomial pneumonia (e.g., HAP, VAP). Nosocomial pneumonia was defined as occurring within 48 hours of hospitalization in accordance with the ATS/IDSA definitions [3]. We only included persons with pneumonia and laboratory confirmation of a bacterial aetiology. Presence of a bacterial aetiology was determined based on a review of electronic health records for culture results (blood, respiratory secretions, pleural fluid) and urinary antigens for either *S*. *pneumoniae* or *Legionella spp*. We only reviewed cultures obtained within 48 hours of admission. The database contains only de-identified data and is HIPAA compliant. Because of this and the nature of the present analysis (review of existing records), this study was classified as exempt from Institutional Review Board (IRB) review.

### Endpoints and covariates

The isolation of MRSA served as our primary endpoint. We compared those with MRSA to those infected with other bacterial organisms. Patients with MRSA isolated along with another pathogen were counted as MRSA for the purposes of this study. Polymicrobial status (more than one isolate) was captured as a separate variable in the study. The determination of methicillin-resistance was made at each individual site in accordance with local laboratory practices. We collected information regarding patient demographics, co morbidities (based on ICD-9-CM codes), severity of illness, and source of admission. Need for intensive care unit (ICU) admission represented our primary measure of disease severity. We identified patients (based on ICD-9-CM codes) with coronary artery disease (CAD), congestive heart failure (CHF), diabetes mellitus (DM), chronic obstructive pulmonary disease (COPD), stroke, dementia, or chronic kidney disease necessitating haemodialysis (HD). We calculated Charlson Co morbidity scores to determine the extent of co morbidities [13]. We additionally categorized patients as to whether they met criteria for HCAP [3]. We defined HCAP as present if any one of the following criteria were met: nursing home, long term acute care facility, or skilled nursing facility (NH/LTC/SNF) exposure within the last 90 days, recent hospitalization in the last 90 days with length of stay ≥ 2 days, prior intravenous antibiotic therapy or wound care in the last 30 days prior to admission, chronic HD, or a history of immunosuppression. Chronic HD persons were identified as those who have undergone dialysis at any setting plus a diagnosis of chronic kidney disease (based on ICD-9-CM codes) within 30 days prior to the index admission or during index admission. Based on clinical expertise, we classified immunosuppressed persons as those who had: 1) recent or current treatment with systemic corticosteroids or other immunosuppressive/chemotherapy agents; 2) a diagnosis of human immunodeficiency disease (HIV), leukaemia/lymphoma, or metastatic cancer, or 3) had undergone a solid/liquid organ transplant. All patients not fulfilling the HCAP definition were categorized as CAP.

### Statistics and risk score development

For continuous variables, MRSA and non-MRSA patients were compared with either the Student’s t-test or Wilcoxon 2-sample test, as appropriate. Categorical data were analyzed with either the chi-square test or the Fisher’s exact test. We defined a p-value of <0.05 to represent statistical significance.

For risk score development and validation, we employed a split sample approach. The entire population was randomly split into a development cohort (two-thirds of patients) and a validation cohort (remaining one-third of patients). We then utilized a bootstrapping based, stepwise logistic regression procedure on the development cohort to determine variables most strongly associated with MRSA [14]. We also assessed plausible interaction terms between various co morbidities as well as co morbidities and gender in a series of models. In each stepwise regression model, a p-value of 0.05 was required for entry into the model and a p-value of 0.1 to remain in the model. The stepwise algorithm was applied to 100 bootstrapped samples in the development set and each time the variable entered the model, this was recorded. Variables that consistently entered the model were kept for further analysis. The final 8 variables that comprised the risk score were those that entered the samples most often and had face validity for clinical relevance.

In the final risk score the standardized coefficients from the logistic regression represented the weight assigned to each predictor variable. Specifically, we fully standardized the coefficients of each variable in the final logistic regression model (i.e., they were standardized on the X and Y variables). Based on the standardized coefficients, we rounded up to get integer “point values” for the score with the smallest standardized coefficients getting a point value of 1 for ease of computation at the bedside. We then validated the risk score in the validation cohort, determining the prevalence of MRSA as a function of the score. We also examined traditional model performance characteristics (e.g., the area under the receiver operating characteristic curve [AUROC]).

## Results

The final study population (both development and validation cohorts) included 5975 patients. MRSA occurred in 14.0% (n = 837) of patients. Other commonly recovered pathogens included: *S*. *pneumoniae* (17.5%), *P*. *aeruginosa* (13.8%), methicillin-sensitive *S*. *aureus* (13.7%), *Escherichia coli* (6.0%), and *H*. *influenzae* (5.6%). As Table 1 demonstrates, in the overall sample patients with MRSA were older and suffered from more co morbidities. Every chronic condition of interest, other than malignancy, occurred more often in patients with MRSA compared to those patients without MRSA. Reflecting this, the Charlson Co morbidity score [mean, standard deviation (SD)] was higher among patients with MRSA compared to no MRSA [(2.6, 2.2) versus (2.2, 2.1), p < 0.001]. A significant portion of MRSA versus no MRSA patients had an ICU admission on or before their index culture (22.9 versus 14.9%, p < 0.001).

**Table 1:** Patient characteristics

	**MRSA**	**No MRSA**	**p**
	**(n = 837)**	**(n = 5138)**	
**Demographics**			
Age, mean ± SD, years	69.4 ± 17.1	67.1 ± 17.0	<0.001
Male, %	53.4%	55.7%	0.435
Race			0.606
Caucasian, %	80.6%	82.1%	
African-American, %	14.6%	13.4%	
Other/Unknown, %	4.8%	4.5%	
**Co morbid illnesses (current or prior to index admission unless noted)**			
Hypertension, %	56.2%	52.8%	0.075
Diabetes mellitus, %	28.0%	25.4%	0.117
Coronary artery disease, %	29.2%	27.6%	0.358
Congestive heart failure, %	35.0%	26.8%	<0.001
Chronic obstructive pulmonary disease, %	50.1%	44.7%	0.004
Malignancy (includes lymphoma, myeloma, secondary neoplasm), %	13.7%	14.%	0.433
Cerebrovascular disease (any) prior to index admission, %	10.6%	5.0%	<0.001
Ischemic stroke prior to index admission, %	5.0%	3.2%	0.007
Dementia, %	9.3%	4.6%	<0.001
Chronic haemodialysis, %	21.1%	15.6%	<0.001
**Healthcare**-**associated risk factors**			
Nursing home/skilled nursing facility/long term acute care exposure within last 90 days, %	25.9%	11.4%	<0.001
Hospital-based wound care within 30 days prior to index admission, %	5.38%	1.32%	<0.001
Recent hospitalization (for ≥ 2 days and within the last 90 days), %	38.7%	21.7%	<0.001
Prior intravenous antibiotic therapy within the last 30 days, %	5.4%	1.3%	<0.001
Immunosuppression, %	23.4%	16.2%	<0.001
**Severity of illness**			
Intensive care unit admission (on or before index culture), %	22.9%	14.9%	<0.001

**Figure 1:**
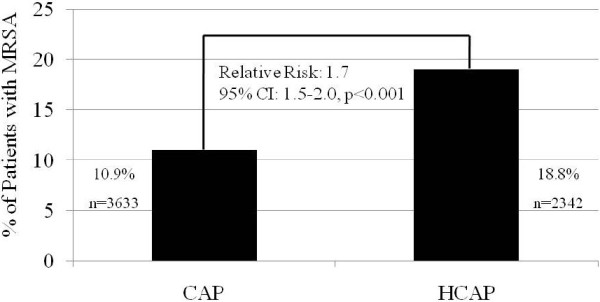
**Prevalence of methicillin-resistant *Staphylococcus aureus *in community-acquired and healthcare**-**associated pneumonia.**

Nearly 40% of those presenting to the hospital with pneumonia met criteria for HCAP, and the prevalence of MRSA was substantially higher in HCAP. Those with HCAP were 1.7 times (95% Confidence Interval (CI): 1.5-2.0) more likely to be infected with MRSA than persons classified as CAP (Figure 1).

**Figure 2:**
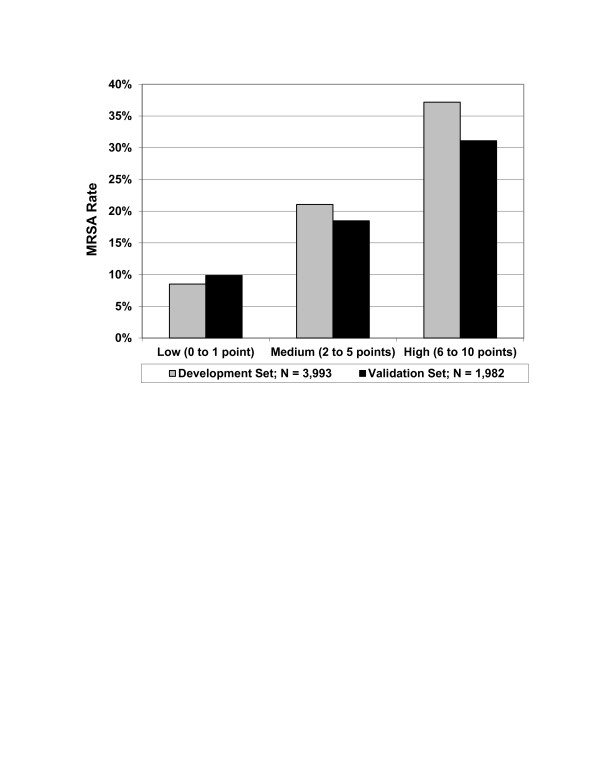
**Prevalence of methicillin-resistant *Staphylococcus aureus *among patients presenting with pneumonia to the hospital as a function of the total risk score.**

The final logistic regression model consisted of eight variables and included demographics, prior healthcare exposure, disease acuity, and select co morbid conditions. Table 2 summarizes the risk score for MRSA pneumonia and the designation of points. Variables more strongly linked with MRSA (based on relative risks) included a recent inpatient hospitalization for ≥ 2 days within the last 90 days and need for ICU admission (see Additional file 3). The HCAP variable was an independent predictor of MRSA but not as strong a predictor as some of its components; therefore, it ultimately was not included in the final model. The total possible score ranged from 0 to 10. Figure 2 shows how the prevalence of MRSA rose with increasing score in both the development and validation cohorts after stratifying the scores into Low (0 to 1), Medium (2 to 5) and High (≥ 6) risk. When the score was ≤1, the prevalence of MRSA was < 10% while the prevalence of MRSA climbed to > 30% when the score was ≥ 6. Of note, the Low Risk subgroup (score of ≤1) represents 57.7% of the overall sample (e.g. both development and validation cohorts). In addition, in the overall sample, 100% of the patients in the High risk group have HCAP versus 18.17% in the Low risk group, p < 0.001.

**Table 2:** **MRSA risk score for methicillin**-**resistant *Staphylococcus aureus *in pneumonia**

**Variable**	**Points**
**Age**	
Age < 30 years or > 79 years	1
**Prior healthcare exposure**	
Recent hospitalization (for ≥ 2 days and within the last 90 days)	2
Nursing home/skilled nursing facility/long term acute care exposure within last 90 days	1
Prior IV antibiotic therapy within the last 30 days	1
**Severity of illness**	
Intensive care unit admission (on or before index culture)	2
**Co morbid illness**	
Cerebrovascular disease (any), prior to admission	1
Dementia	1
Female with diabetes mellitus	1
Possible total point score: 10	

As a screening test, a score of ≤1 versus greater than 1 had a sensitivity and specificity of 59.1% and 60.0% respectively. The positive predictive value was low at 19.2% but the negative predictive value equalled 90.1%. The pre-test probability for MRSA was approximately 14% (e.g., the prevalence of MRSA in the overall cohort). With a ≤1 classification threshold, patients with a score ≤1 would have a post-test (posterior) probability of MRSA of 10% (a reduction in risk of approximately one-third) and the post-test probability of MRSA would be 19.4% for those with scores >1. The AUROCs of this model in the development and validation cohorts were 0.66 (95% CI: 0.63, 0.68) and 0.64 (95% CI: 0.60, 0.67), respectively.

## Discussion

This large retrospective analysis of microbiologically-confirmed culture positive pneumonia patients presenting to the hospital reveals that MRSA constitutes an important pathogen in this setting. Similarly, HCAP accounts for a large proportion of all pneumonia patients admitted to the hospital and is nearly as common as CAP. Even though patients with HCAP face an increased risk for MRSA, the prevalence of MRSA in HCAP was less than 20%. A risk scoring tool based on factors independently associated with the recovery of MRSA segregates patients with moderate accuracy. A low total score describes a cohort of individuals unlikely to have MRSA and in whom anti-MRSA therapy can likely be withheld.

Our data add to the growing evidence elucidating the frequency of HCAP relative to CAP. In the initial study examining the epidemiology of HCAP, HCAP accounted for approximately one-third of all pneumonias presenting to the emergency department (ED) [15]. Other reports have documented that HCAP represents between 30% and 60% of pneumonias initially evaluated in the ED [5-7], and this trend is also observed in Asia and Europe [9,16]. Our study confirms that MRSA is a common pathogen in patients presenting to the hospital with their pulmonary infections. Classically thought only to be significant in nosocomial infections, it is now evident that physicians treating those who come to the hospital with pneumonia must consider MRSA when selecting antibiotic regimens.

Originally, the notion of HCAP was developed to help with this decision making process [3]. The specific components of the HCAP definition were derived from expert opinion and were initially adopted from a single centre study in bacteraemia [3,17]. HCAP was meant to serve as a tool to help clinicians stratify individuals as to the chance that a resistant pathogen, like MRSA, was a practical issue. Our finding that there are many patients with HCAP who are not infected with MRSA suggests that HCAP alone cannot serve as a precise tool for risk stratification. Our findings further confirm the observations of earlier researchers. For example, Schrieber and colleagues found that HCAP performed poorly as a screening test for MRSA in persons presenting to the hospital with severe pneumonia necessitating mechanical ventilation [6]. Shorr et al. similarly found that HCAP misclassified many patients both as to their risk for resistant pathogens in general, and for MRSA, specifically [5]. Thus, it appears that broad use of the HCAP criteria for classification can lead to overuse of broad spectrum antibiotics which can result in unnecessary cost and antimicrobial resistance.

The proposed risk score is novel and improves on the status quo, as it provides a means for reliably classifying quickly and easily a large cohort of patients at low risk for MRSA. Since anti-MRSA therapies can often be added or withheld without necessarily adjusting the selected Gram-negative coverage, having an effective way to limit the prescription of anti-MRSA treatments becomes crucial. The situation with respect to anti-MRSA treatment options is even more acute given their costs and potential side effects.

Prior reports have proposed alternate risk scoring schemes for determining the probability of resistant organisms in those coming to the hospital with pneumonia [5-7]. However, most of these studies have pooled resistant Gram negative organisms (e.g., *P*. *aeruginosa*) with MRSA [5-7]. Our effort is therefore unique in that we have examined MRSA specifically. Our results are also novel in that we identify several factors associated with MRSA that have not previously been linked to pneumonia with this pathogen. For example, extremes of age have not been previously described as related to MRSA in pneumonia. Likewise the description of a nexus between certain co morbid diseases, such as cerebrovascular disease (CVD) and dementia, with MRSA has not been noted before in evaluations of pneumonia in the ED. The mechanism of these associations with MRSA is unclear. These specific factors might actually represent surrogates for other factors that we could neither address nor measure in the dataset. Additionally, it appears that not all factors contribute equally to the risk for MRSA as a cause of pneumonia on hospital presentation. Intense exposure to the healthcare system (e.g., recent inpatient stay) seems disproportionately important as does severity of illness. Moreover, different risk scores utilized thus far in pneumonia (e.g., CURB-65) focus solely on severity of illness while our risk score specifically deals with issues of aetiology.

Our study has several strengths. It is based on a large sample from multiple hospitals across the US. This provides us the power to examine select conditions that other analyses could not evaluate because of their smaller size. It also suggests the generalizability of our findings. We have also internally validated the proposed risk score. No other effort has applied such a split sample approach in pneumonia presenting to the hospital. In fact, many risk stratification schemes for other disease states never undergo any effort at either internal or external validation.

Conversely, the present investigation has a number of significant limitations. First, its retrospective nature exposes it to several forms of bias. This is particularly concerning since we relied on ICD-9-CM coding to identify patients with pneumonia or a primary diagnosis of sepsis with a secondary diagnosis of pneumonia. There certainly are patients we included who likely had some alternate diagnosis. Second, culture data are not always obtained in every case of suspected pneumonia which introduces a selection bias. A priori, physicians may have preferentially opted to obtain cultures in patients when they were already concerned about resistant pathogens. Moreover, cultures can be negative even in the presence of active bacterial infection and positive cultures of the respiratory tract can represent colonization and not true infection. Again, this raises the concern that we could have misclassified patients. On the other hand, since we only studied culture positive patients, our findings likely underestimate the potential for antibiotic overuse and abuse. Third, we did not evaluate patients for co-infection with viral pathogens which could have influenced our findings, especially as a risk factor for MRSA infection. Fourth, despite our attempt to evaluate all components of the HCAP definition, we likely miscategorised some patients because of inadequate information regarding recent IV antibiotic exposure since administrative datasets may not consistently capture this. Some of the factors identified, such as dementia, could represent surrogates for other characteristics about which we lacked data. Finally, our score had only moderate discriminatory power and requires external validation. Although it identified a group of persons as low risk for MRSA, our data illustrate that there is a clear need for better tools for this purpose and that we urgently require rapid diagnostic tests for organism identification.

## Conclusions

MRSA represents a cause of pneumonia presenting to the hospital. A simple risk score for identifying MRSA in patients presenting to the hospital with pneumonia identifies those at low risk for MRSA and may facilitate efforts to better direct antibiotic prescribing.

## Abbreviations

AUROC: Area under the receiver operating curve; CVD: Cerebrovascular disease; COPD: Chronic obstructive pulmonary disease; CAP: Community-acquired pneumonia; CI: Confidence interval; CHF: Congestive heart failure; CAD: Coronary artery disease; DM: Diabetes mellitus; ED: Emergency department; HCAP: Healthcare-associated pneumonia; HIPAA: Health insurance portability and accountability act; HD: Haemodialysis; HAP: Hospital-acquired pneumonia; HIV: Human immunodeficiency virus; ICU: Intensive care unit; ICD-9-CM: International Classification of Diseases, 9^th^ Revision, Clinical Modification; IRB: Institutional review board; LTC: Long term care facility; MRSA: Methicillin resistant *Staphylococcus aureus*; NH: Nursing home; SNF: Skilled nursing facility; SD: Standard deviation; US: United States; VAP: Ventilator-associated pneumonia.

## Competing interests

Dr. Shorr has served as a consultant to, speaker for, or received grant support from Astellas, Bayer, Forrest, Pfizer, Theravance, and Trius. Ms. Myers is an employee and shareholder of Pfizer Inc and Dr. Huang was an employee and shareholder of Pfizer Inc at the time this study was conducted. Dr. Nathanson is an employee of OptiStatim, LLC which has a consulting agreement with Cerner. Dr. Emons is an employee of Cerner Corporation, which has performed consulting services for Pfizer and other pharmaceutical companies. Dr. Kollef has served as a consultant, speaker for, or received grant support from Cubist, Hospria, Merck, and Sage.

## Authors’ contributions

AFS participated in the study concept and design, drafted the manuscript, and helped supervise the study. DEM participated in the study concept and design, obtained funding, and helped supervise the study. DBH participated in the concept and design. BHN helped acquire the data and provided statistical expertise. MFE participated in the study concept and design and helped acquire the data. MHK participated in the study concept and design and contributed to drafting the manuscript. All authors contributed to the analysis and interpretation of the data as well as the critical revision of the manuscript for important intellectual content. All authors read and approved the final manuscript.

## Pre-publication history

The pre-publication history for this paper can be accessed here:

http://www.biomedcentral.com/1471-2334/13/268/prepub

## Supplementary Material

Additional file 1 **Additional details about *Health Facts *database.** Click here for file

Additional file 2 ICD-9-CM codes. Click here for file

Additional file 3 Relative risk of MRSA based on development set. Click here for file
