# Supplementary material for: A risk score for identifying methicillin-resistant Staphylococcus aureus in patients presenting to the hospital with pneumonia
Source: BMC Infect Dis. 2013 Jun 6;13:268. doi: 10.1186/1471-2334-13-268 (PMC3681572; doi:10.1186/1471-2334-13-268)
Supplement: Additional file 1 — Additional details about Health Facts database. [file 1471-2334-13-268-S1.pdf]

Additional details about *Health Facts*

The *Health Facts* database is not a public database. Cerner Corporation licenses the data and makes it available for research purposes, including industry-sponsored and academic research. Research with the data is conducted under the terms of a data use agreement, which includes provisions to protect the de-identified nature of the data. Cerner researchers and analysts were active collaborators on this project and conducted all analyses themselves.

Health Facts is a large database containing clinical, laboratory, and administrative data from over 60 hospitals in the US. Content is drawn from electronic health records. All census regions are represented. Unlike the National Inpatient Sample, *Health Facts* is not specifically designed to be a nationally representative sample of hospital discharges. However, all US Census Regions are represented and there is a good blend of hospital bed size by category. The overwhelming majority of hospitals are urban. About two-thirds are teaching hospitals.
