# Supplementary material for: A risk score for identifying methicillin-resistant Staphylococcus aureus in patients presenting to the hospital with pneumonia
Source: BMC Infect Dis. 2013 Jun 6;13:268. doi: 10.1186/1471-2334-13-268 (PMC3681572; doi:10.1186/1471-2334-13-268)
Supplement: Additional file 2 — ICD-9-CM codes. [file 1471-2334-13-268-S2.pdf]

ICD-9-CM codes

The definition of pneumonia based on primary discharge diagnosis with any of the following:

ICD-9-CM diagnosis	Description
481	Pneumococcal pneumonia (*streptococcus pneumoniae* pneumonia)
482.xx	Other bacterial pneumonia
483.x	Pneumonia due to other specified organism
484.3	Pneumonia in whooping cough
484.5	Pneumonia in anthrax
485	Bronchopneumonia organism unspecified
486	Pneumonia organism unspecified
487.0	Influenza with pneumonia
507.0	Pneumonitis due to inhalation of food or vomitus

We chose to omit fungal infections, viral infections other than influenza and did not include lung abscess or empyema since these should not be used alone without a diagnosis of pneumonia.

For the diagnosis of sepsis the below ICD-9-CM diagnosis codes were utilized.

ICD-9-CM diagnosis	Description
038.xx	Septicemia
040.82	Toxic shock syndrome

785.52	Septic shock
995.91*	Systemic inflammatory response syndrome due to infectious process without organ dysfunction
995.92*	Systemic inflammatory response syndrome due to infectious process with organ dysfunction

* ICD-9-CM code became effective in January 2003
