# Supplementary material for: A risk score for identifying methicillin-resistant Staphylococcus aureus in patients presenting to the hospital with pneumonia
Source: BMC Infect Dis. 2013 Jun 6;13:268. doi: 10.1186/1471-2334-13-268 (PMC3681572; doi:10.1186/1471-2334-13-268)
Supplement: Additional file 3 — Relative risk of MRSA based on development set. [file 1471-2334-13-268-S3.pdf]

Variable	Relative Risk based on the Development Set	95% CI	P-Value
**Age**			
Age < 30 years or > 79 years	1.183	(1.001, 1.391)	0.049
**Prior Healthcare Exposure**			
Recent hospitalization (for ≥ 2 days and within the last 90 days)	1.556	(1.291, 1.859)	<0.001
Nursing home/skilled nursing facility/long term acute care exposure within last 90 days	1.509	(1.212, 1.859)	<0.001
Prior intravenous antibiotic therapy within the last 30 days	1.619	(1.075, 2.340)	0.022
**Severity of Illness**			
Intensive Care Unit Admission (on or before index culture)	1.652	(1.380, 1.961)	<0.001
**Comorbid Illness**			
Cerebrovascular Disease (any), prior to admission	1.233	(0.920, 1.628)	0.159
Dementia	1.548	(1.160, 2.024)	0.003
Female with diabetes mellitus	1.327	(1.071, 1.630)	0.010
